# Pu-Erh Tea Down-Regulates Sterol Regulatory Element-Binding Protein and Stearyol-CoA Desaturase to Reduce Fat Storage in *Caenorhaditis elegans*

**DOI:** 10.1371/journal.pone.0113815

**Published:** 2015-02-06

**Authors:** YiHong Ding, XiaoJu Zou, Xue Jiang, JieYu Wu, YuRu Zhang, Dan Chen, Bin Liang

**Affiliations:** 1 Key Laboratory of Animal Models and Human Disease Mechanisms of the Chinese Academy of Sciences & Yunnan province, Kunming Institute of Zoology, Chinese Academy of Sciences, Kunming, 650223, China; 2 Department of Life Science and Technology, Key Laboratory of Special Biological Resource Development and Utilization of University in Yunnan Province, Kunming University, Kunming, 650214, China; The University of New South Wales, AUSTRALIA

## Abstract

Consumption of Pu-erh has been reported to result in numerous health benefits, but the mechanisms underlying purported weight-loss and lowering of lipid are poorly understood. Here, we used the nematode *Caenorhaditis elegans* to explore the water extract of Pu-erh tea (PTE) functions to reduce fat storage. We found that PTE down-regulates the expression of the master fat regulator SBP-1, a homologue of sterol regulatory element binding protein (SREBP) and its target stearoyl-CoA desaturase (SCD), a key enzyme in fat biosynthesis, leading to an increased ratio of stearic acid (C18:0) to oleic acid (C18:1n-9), and subsequently decreased fat storage. We also found that both the pharyngeal pumping rate and food uptake of *C. elegans* decreased with exposure to PTE. Collectively, these results provide an experimental basis for explaining the ability of Pu-erh tea in promoting inhibition of food uptake and the biosynthesis of fat via SBP-1 and SCD, thereby reducing fat storage.

## Introduction

Obesity is an outcome of energy intake exceeding energy expenditure, which leads to the extra energy becoming stored mainly as fat due to abnormal proliferation and over excessive volume of adipocytes. Globally, obesity and its comorbidities, type 2 diabetes, cardiovascular diseases, non-alcoholic fatty liver disease, among others, are increasingly prevalent, and present a rising public health concern, especially to the ∼1.46 billion overweight and the ∼502 million obese adults[1]. Given the alarming rise in these trends in both developed and developing countries, there is a stark need for more effective therapies that can both prevent and treat obesity and obesity-related diseases. One potential such therapy may lie in a long used traditional beverage produced in southwestern China known as Pu-erh tea. Pu-erh tea, a tea post-fermented by microorganisms[2] has long been consumed for its supposed benefits to health that are only recently being scientific explored, marked effects against oxidation [3–5], cancer[6], atherosclerosis [7], obesity[8,9], hypercholesterolemia, [10–12], among others.

Despite great progress in both *in vivo* and *in vitro* researches into the effects of Pu-erh tea on human, rodents, and cells to ability to reduce body weight, fat mass, and the level of serum triglycerides, cholesterol, and low-density lipoproteins (LDL) while also inducing the level of high-density lipoproteins, and the progression of steatosis[7–9,13–17]. Unfortunately, the mechanisms by which Pu-erh tea consumption lowers body fat and lipid profiles is poorly understood. Over the past decade, however, the nematode *C*. *elegans* has become an increasingly popular model for investigating the regulation of fat metabolism and obesity genetics[18–23], since *C*. *elegans* is quite similar to other organisms in its storage fat in lipid droplets[18,24–26]. In the present study, we used this model to explore the underlying mechanisms of Pu-erh tea’s weight-cutting effects. First, we investigated the effect of Pu-erh tea on fat storage reduction using Nile Red staining [26], and quantitation via thin layer chromatography and gas chromatography (TLC/GC), followed by food uptake and absorption We also explored the effects of Pu-erh tea on stearoyl CoA desaturase (SCD), a key enzyme in the biosynthesis of fat, and its upstream regulator SREBP[27,28].

## Materials and Methods

### Quantification of the water extract of Pu-erh tea (PTE)

Pu-erh tea (100 g, LongRun Ripen Pu-erh; LongRun Group, Yunnan, China) was soaked in 500 ml boiling water for 20 min, then the water extract of Pu-erh tea (PTE) was centrifuged, filtered and sterilized with a filter membrane (0.45 μm pore size) at room temperature. The composition of PTE is surprisingly complex, with polyphenols, polysaccharides, caffeine, protein, and amino acids at low concentrations[29] Consequently, quantifying the concentration of PTE based on one specific compound can present some difficulties. Here, we used a microplate reader (Biotek, Synergy H1) and found that the full wavelength absorbance of PTE displayed a peak at 590 nm (S1A Fig.) wavelength with absorbance, and also the absorbance OD of PTE presented a linear relationship (R^2^ = 0.9155) (S1B Fig.) with dilution. Accordingly, we set the absorbance of PTE at 590 nm as standard to make nematode growth medium (NGM) plates with the corresponding concentrations of PTE. Precisely which compound in PTE results in the peak at 590 nm wavelength is not clear, as PTE contains a large body of tea pigments that may contribute. Nevertheless, this simple method using a microplate reader helped us to control the concentrations of PTE in this study. The quantified PTE (0.2 g/ml) was added and mixed with nematode growth medium (NGM) before pouring the plates to the final concentrations of 0 g/ml, 0.0125 g/ml, 0.025 g/ml, and 0.05 g/ml (S1C Fig.). All prepared plates with PTE were used in 2 days.

### Worm strains and maintenance

*C*. *elegans* were grown on nematode growth media (NGM) with OP50 strain of *E*. *coli* as a food source. The wild-type strain was N2. The following mutant strains were analyzed: *sbp-1(ep79)*[30], *fat-6(tm331);fat-7(wa37)*[31], *fat-6*::*GFP{BX115*, *lin-15(n765)X;waEx16[fat-6WG*::*GFP];lin-15(+)}*[32], *fat-7*::*GFP{BX113*, *lin-15(n765)X;waEx15[fat-7WG*::*GFP;lin-15(+)])}*[32], *KQ377(N2; ftISf [epEx307[unc-119(+); pSbp-1*:*SBP-1*:*GFP])*, *daf-1(e1370)*, *age-1(hx546)*, *akt-1(mg144)*, and *aak-2(gt33)*. Generally, synchronized L1 worms were placed on NGM plates with different concentrations of PTE, and were cultured under standard conditions.

### Lipids extraction and analysis

Young adult worms with 1–3 eggs were washed off growing plates and immediately frozen in liquid nitrogen and stored at -80°C freezer until further use. To determine the levels of triacylglycerol (TAG) and phospholipids, lipid extraction and separation, we performed thin-layer chromatography (TLC) according to previously published protocols[31,33,34]. Fatty acid compositions of adult worms was determined by gas chromatography (GC) as previously described[26,30–32,34,35].

### Lipid droplets visualization by Nile Red staining of fixed nematodes

Late L4s and young adults worms with 1–2 eggs were washed off growing plates, fixed and stained with Nile Red as described previously[26,30,34]. Images were captured using identical settings and exposure time for each image. Quantification of lipid droplet size was done as described previously[34].

### Pumping rate of pharynx

The pumping rate of the pharynx was measured among young gravid adults growing on OP50 bacteria lawn at 20°C. Pumping rate was measured for each animal by counting the rhythmic contractions of the pharyngeal bulb over a 20 s period under microscope. All measurements were done at room temperature (22°C ± 1°C).

### Feeding *C*. *elegans* with *E*. *coli* transformed with GFP

*E*.*coli* XL1-Blue transformed with *gfp* gene was an indirect gift as previously described[36]. Synchronized L1 N2 worms were placed on NGM plates fed with *E*. *coli* XL1-Blue with different concentrations of PTE, and then cultured under standard conditions. Next, late L4 worms were picked and placed on agarose pad, and then visualizes under a fluorescent microscope. Images were captured using identical settings and exposure time was 428 ms for each of the tested worms.

### Visualization of SBP-1::GFP, FAT-6::GFP, and FAT-7::GFP

Totally, 5–6 adults of *fat-6*::*GFP*, *fat-7*::*GFP*, and *sbp-1*::*GFP* were picked and transferred to new NGM plates with different concentrations of PTE, and were then cultured under standard conditions. Roughly 3 d late, the next generation of late L4 worms were picked and placed on agarose pad, and visualized under a fluorescent microscope. The exposure time for the *fat-6*::*GFP* worms was 180 ms, for the *fat-7*::*GFP* worms was 250 ms, for the *sbp-1*::*GFP* worms was 800 ms.

### Brood size, growth rate and lifespan analysis

Typical brood size, growth rate, and lifespan analysis of *C*. *elegans* were previously described in several studies[26,30,31]. Here, lifespan analysis was carried out at 20°C. L4 worms were transferred to fresh plates with or without PTE at the beginning of the experiment. Worms were transferred to fresh plates daily until they stopped laying eggs, after which they were transferred every 3–4 days. Lifespans were finally listed as the average of three to four independent trials, each using 80–100 animals.

### Quantitative RT–PCR (QPCR) analysis

Quantitative RT-PCR (QPCR) was done in accordance with previously published protocol[30]. In brief, L4s worms were harvested and total RNA was prepared using TransZol Up (Code#ET111–01, TransGen Biotech, Beijing), 2 μg of total RNA was used in a reverse-transcription reaction with PrimeScript RT reagent Kit (Cat#RR047A, Takara Bio Inc. Japan) to generate cDNA. Primer sequences for the lipid metabolism genes were obtained from Marc Van Gilst[10]. The PCR mixture consisted of 0.2 μM primers, cDNA, ROX, and 1× SYBR green mix (TransStart TipTop Green qPCR SuperMix, Cat#AQ141, TransGen Biotech, Beijing). QPCR) was run and monitored on an ABI 7900HT analyzer (Applied Biosystems, Foster City, CA). Relative abundance was determined using the ΔΔCt method and the reference genes *act-1* was used as a control for template levels.

### Statistics and preparation of figures

Statistical analysis was performed using t-test or analysis of variance (ANOVA) followed by LSD multiple comparison test by SPSS 20.0 (SPSS Inc., Chicago, IL, USA), and all figures were made using PrismGraph5.

## Results

### PTE reduced fat storage and lipid droplet size

**Fig 1:**
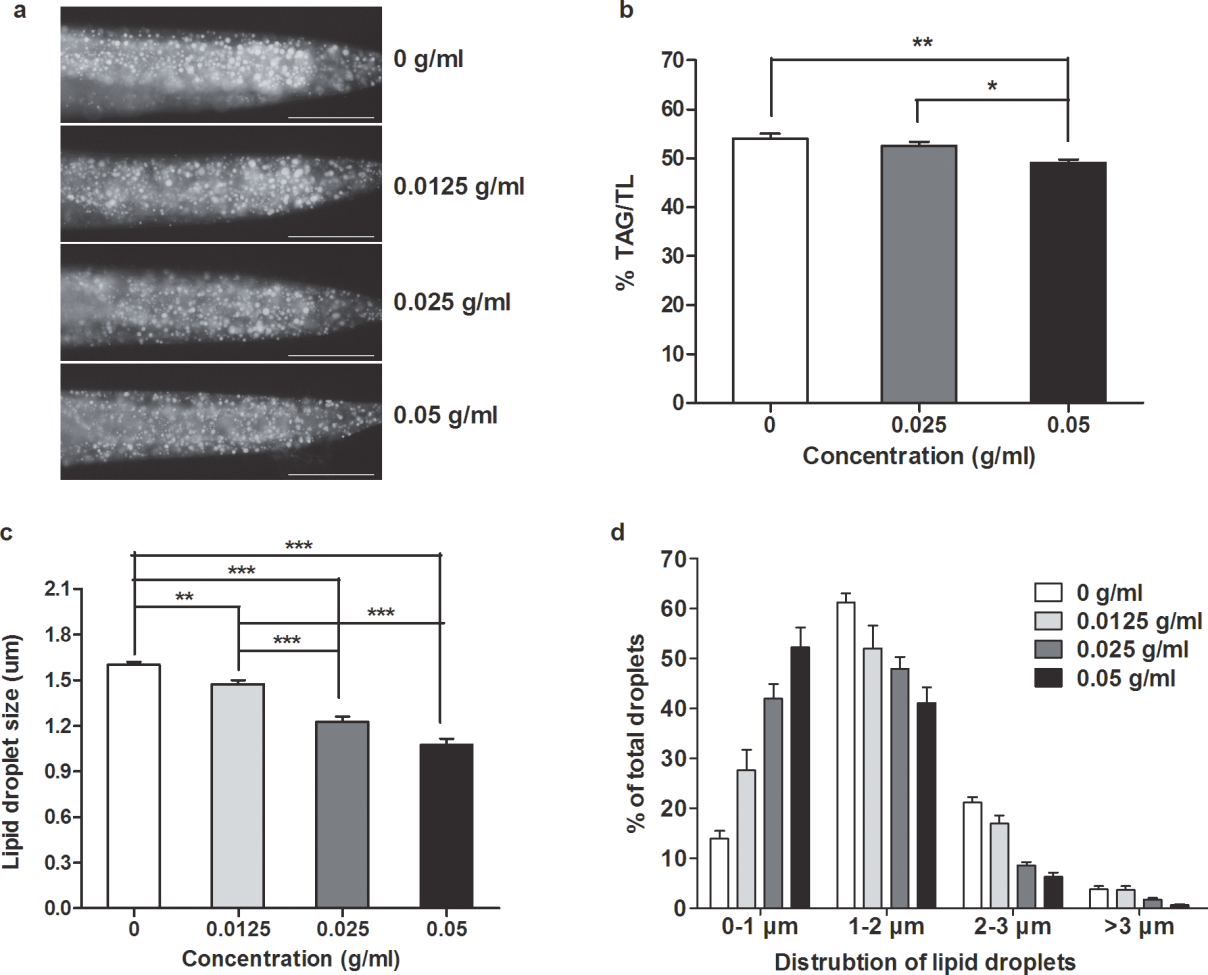
PTE reduced fat storage and lipid droplet size. (a) Fat storage indicated by Nile Red staining of fixed late L4 N2 worms treated with different concentrations of PTE. The anterior is in left and the posterior is in the right (Representative sample of around 50 worms totally observed in three biological replicates for each PTE treatment). Scale bar is 50 μm. (b) Relative TAG (triacylglycerol) content in total lipids (triacylglycerol+phospholipids, % TAG/TL) determined by TLC/GC. TAG content was significantly decreased at 0.05 g/ml concentration of PTE (**: P<0.01, *: P<0.05). Data were the mean ± SEM of four independent biological replicates. (c) Quantitation of the lipid droplet size. Lipid droplet size was correspondingly decreased with the increased concentrations of PTE (**: P<0.01, ***: P<0.001). Data were the mean ± SEM of 10 worms. (d) Distribution of lipid droplets. PTE gradually increased the percentage of small size lipid droplets (0–1 um in diameter), but decreased the percentage of large size lipid droplets (>1um in diameter). Data were the mean ± SEM of 10 worms.

We raised *C*. *elegans* wild type N2 worms on NGM plate with different concentrations of PTE to trace the effects of PTE on fat storage. Compared with the control (0 g/ml), Nile Red staining indicated that fat storage among fixed worms gradually decreased with the increased concentrations of PTE (Fig. 1A). Concurrently, worm fat contents (% TAG/TL) measured by TLC/GC decreased with the increased concentrations of PTE. The percent of TAG content of the control worms was 54.05%, whereas it was significantly reduced to 49.09% when treated with the highest concentration of Pu-erh tea (0.05 g/ml) (P<0.001) (Fig. 1B), indicating a reduction of fat content of 9.17%. The average size of lipid droplets in N2 worms was 1.60 ± 0.02 um (Mean ± SEM, n = 10 (Fig. 1C), but administration of PTE gradually (though significantly) decreased the size of lipid droplets to 1.47 ± 0.03 um (n = 10, P<0.01), 1.21 ± 0.03 um (n = 10, P<0.001), and 1.07 ± 0.04 um (n = 10, P<0.001) when treated with 0.0125 g/ml, 0.025 g/ml, and 0.05 g/ml, respectively (Fig. 1C). Additionally, increased concentrations of PTE led to a change in the distribution of lipid droplets with the portion of the small size lipid droplets (0–1.0 um in diameter) increasing while the large size lipid droplets (>1.0 um in diameter) decreasing (Fig. 1D). Collectively, these results demonstrated that PTE showed a marked effect on reduction of fat storage and lipid droplet size in the *C*. *elegans* model.

### PTE modified the level of fatty acid compositions

**Fig 2:**
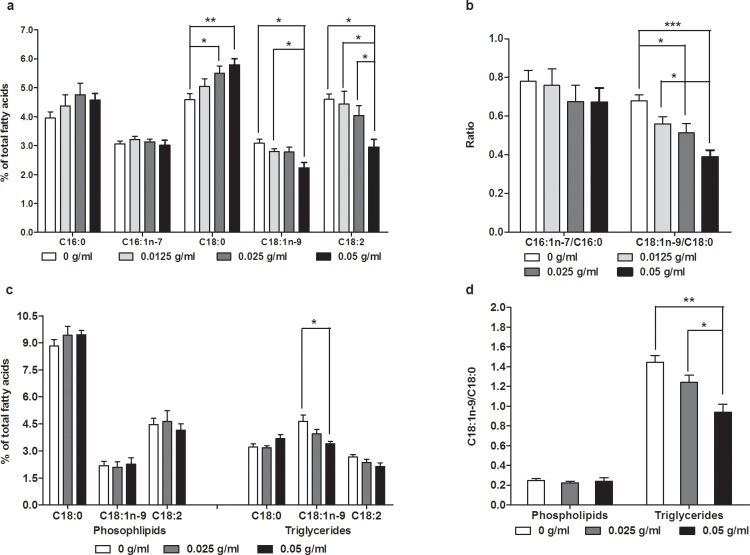
PTE modified the level of fatty acid compositions. PTE significantly decreased the levels of oleic acid (C18:1n-9) and linolenic acid (C18:2), but increased the level of stearic acid (C18:0) (a), decreased the ratio of C18:1n-9/C18:0 (b), changed the levels of oleic acid (C18:1n-9) (c) as well as the ratio of C18:1n-9/C18:0 only in triglycerides (d). Data were presented as mean ± SEM of four independent biological replicates. (*: P<0.05, **: P<0.01, ***: P<0.001).

Fatty acids, especially monounsaturated fatty acid palmitoleic acid (C16:1) and oleic acid (C18:1n-9), are important substrates for biosynthesis of triglycerides, phospholipids, cholesterol esters, and the like. Given the results of PTE inhibiting fat accumulation (Fig. 1A and 1B), we were curious as to whether administration of PTE also affects the biosynthesis of fatty acids in a matter that leads to reduced fat content. *C*. *elegans* consists of many fatty acids including saturated fatty acids and polyunsaturated fatty acids[35], and mono-methyl branched fatty acids[37]. Our results showed that PTE slightly, but not significantly increased the level of palmitic acid (C16:0) (Fig. 2A), but significantly increased the level of stearic acid (C18:0), while also reducing the levels of both unsaturated fatty acids oleic acid (C18:1n-9) and linolenic acid (C18:2) (Fig. 2A). Consistently, we observed a ratio of C18:1n-9/C18:0 that decreased with the increasing concentrations of PTE (Fig. 2B).

We also examined whether administration of PTE can also affected the level of fatty acids in phospholipids or triglycerides. First, we separated phospholipids and triglycerides by thin layer chromatography (TLC), and detected the fatty acid compositions by gas chromatography (GC). Results showed that the levels of C18:0, C18:1n-9, and C18:2 were not changed in phospholipids, but the levels of C18:1n-9 and C18:2 slightly decreased with increasing concentrations of PTE in triglycerides (Fig. 2C). Meanwhile, the ratio of C18:1n-9/C18:0 was significantly decreased only in triglycerides, but not in phospholipids (Fig. 2D). These results suggested that PTE inhibited the conversion of saturated fatty acid stearic acid (C18:0) to oleic acid (C18:1n-9), and further to linolenic acid (C18:2) in triglycerides, which might lead to a reduction of fat storage in *C*. *elegans*.

### PTE down-regulated the expression of stearoyl-CoA desaturase and SBP-1

**Fig 3:**
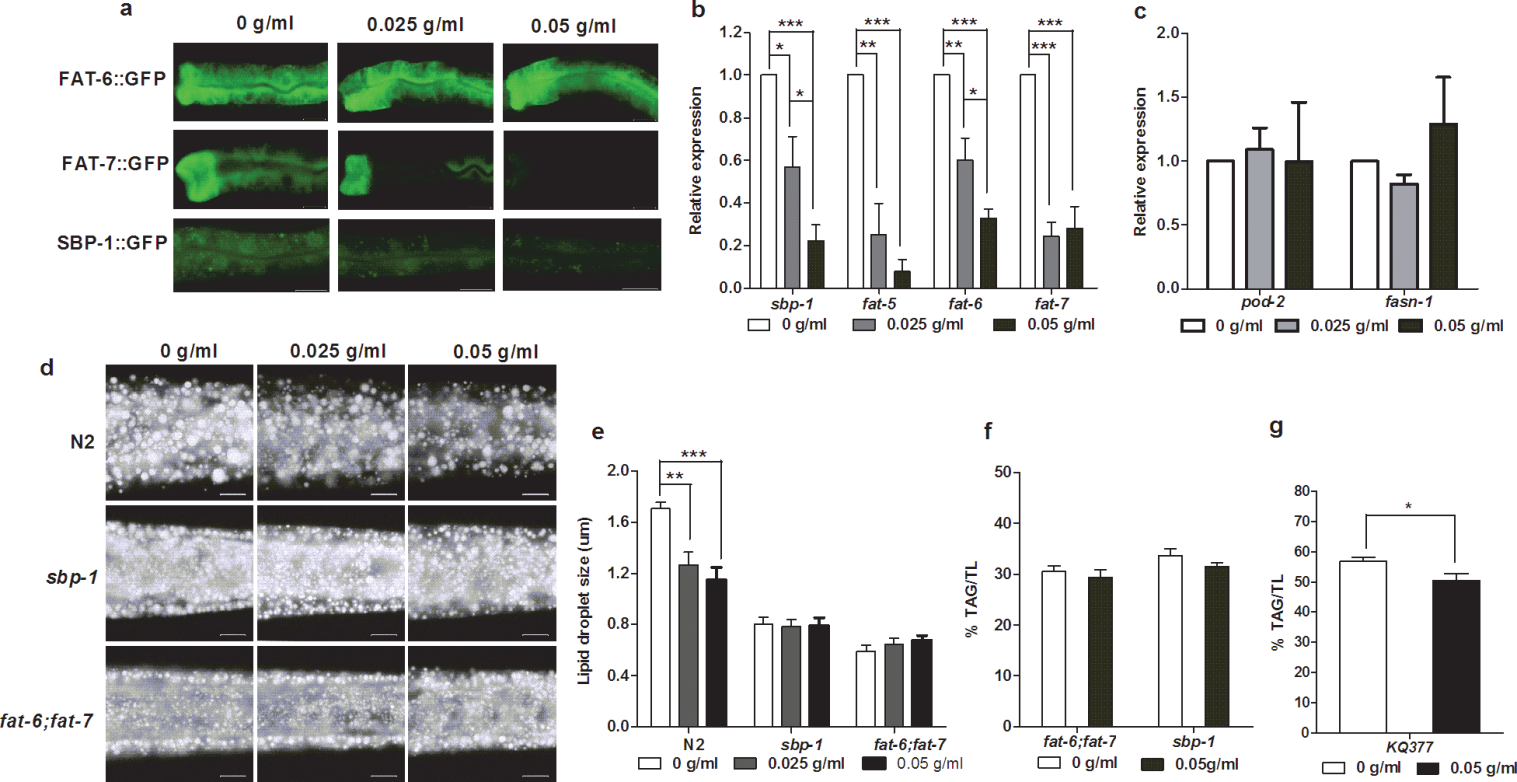
PTE down-regulated the expression of SBP-1 and SCD. (a) PTE did not affect the expression of FAT-6::GFP (around 60 worms totally observed in three biological replicates), but gradually decreased the fluorescent intensity of FAT-7::GFP and SBP-1::GFP (> 80% worms showed consistent reduction of expression, around 60 worms totally observed in three biological replicates). Scale bar is 20 μm. (b) Relative expression levels of *sbp-1*, *fat-5*, *fat-6*, and *fat-7* measured by QPCR were significantly decreased with the increased concentrations of PTE. Data were the mean ± SEM of three independent biological replicates. (*: P<0.05, **: P<0.01, ***: P<0.001). (c) Relative expression levels of *pod-2* and *fasn-1* measured by QPCR. Data were the mean ± SEM of three independent biological replicates. (d) Fat storage indicated by Nile Red staining of fixation was gradually decreased in N2 worms, but not in *sbp-1* and *fat-6;fat-7* worms with the increased concentrations of PTE. The anterior is in left and the posterior is in the right. Scale bar is 10 μm. (e) Lipid droplet size was significantly decreased in N2 worms, but not in *sbp-1* and *fat-6;fat-7* worms, with the increased concentrations of PTE. Data were the mean ± SEM of 6 worms. (**: P<0.01, ***: P<0.001). (f) % of TAG/TL of *fat-6;fat-7* and *sbp-1* mutants treated with PTE. Data were the mean ± SEM of four independent biological replicates. (g) PTE significantly decreased the fat content of *KQ377* worms. Data were the mean ± SEM of four independent biological replicates.

Stearoyl-CoA desaturase (SCD) is a key enzyme that converts saturated fatty acids palmitic acid (C16:0) and stearic acid (C18:0) to palmitoleic acid (C16:1n-7) and oleic acid (C18:1n-9)[38], respectively. As we noted earlier, PTE increased the level of stearic acid (C18:0) (Fig. 2A) while also decreased the level of oleic acid (C18:1 n9) (Fig. 2A and 2C) and the ratio of C18:1n-9/C18:0 (Fig. 2B and 2D). We accordingly investigated the effect of PTE on SCD. Totally, *C*. *elegans* genome contains three *scd* genes, *fat-5*, *fat-6*, and *fat-7*[31,32], with FAT-5 mainly converting palmitic acid (C16:0) to palmitoleic acid (C16:1n-7), and both FAT-6 and FAT-7 converting stearic acid (C18:0) to oleic acid (C18:1n-9)[39]. We treated *fat-6*::*GFP* and *fat-7*::*GFP* transgenic worms[32] with different concentrations of PTE, and noticed that while the fluorescence intensity of FAT-6::GFP was not changed with increased concentrations of PTE, that of FAT-7::GFP dramatically decreased (Representative samples were shown in Fig. 3A). QPCR indicated that the expression of all three *scd* genes, *fat-5*, *fat-6*, and *fat-7*, were significantly decreased by administration of PTE as compared to the control worms (Fig. 3B).

Sterol regulatory element binding protein (SREBP) is an important transcription factor regulating fatty acids, cholesterol, and other lipids metabolism[27,28]. SCD is a key target of SREBP[20,30,40–42]. To determine whether PTE affected the expression of *fat-5*, *fat-6*, and *fat-7* depended on SBP-1, a homolog of SREBP in *C*. *elegans*, we treated *sbp-1*::*GFP* (*KQ377*) worms, an integrated strain of *Psbp-1*:*sbp-1*:*gfp* in WT (see Material and Methods as well as ref.[43]), with different concentrations of PTE. Like FAT-7::GFP, the fluorescence intensity of SBP-1::GFP was correspondingly decreased with the increased concentrations of PTE (Representative sample was shown in Fig. 3A). Additionally, the mRNA level of *sbp-1* determined by QPCR was also reduced by PTE (Fig. 3B). By contrast, the expression of *pod-2* and *fans-1*, which encode acetyl-CoA carboxylase and fatty acid synthase that are predicted to catalyze the first and second step in *de novo* fatty acid biosynthesis in *C*. *elegans*, was not changed by administration of PTE (Fig. 3C).

### Mutation of *sbp-1* and *fat-6;fat-7* abolished the reduced fat storage by PTE

Since PTE down-regulated the expression of *sbp-1*, *fat-6*, and *fat-7*, and also inhibited the ratio of C18:1n-9/C18:0 in triglyceride, we further sought to clarify whether mutation of either *sbp-1* or *fat-6*;*fat-7* could abrogate the effect of reduced fat storage by PTE. In wild type N2 worms, PTE significantly decreased fat storage (Fig. 3D) and the size of lipid droplets (Fig. 3E), consistence with previous reports (Fig. 1A, 1B and 1C). Conversely, *sbp-1* and *fat-6;fat-7* mutants, which have low fat storage and small size of lipid droplets compared to N2 worms[30,31], did not response to PTE and neither fat storage (Fig. 3D) nor lipid droplet size (Fig. 3E) changed. During our earlier experiments we noticed that PTE significantly reduced the fat content quantified by TLC/GC at 0.05 g/ml in WT worms (Fig. 1B), so we opted to use this concentration to treat *fat-6;fat-7* and *sbp-1* mutant worms. Our results, however, showed that administration of PTE left the fat content of *fat-6;fat-7* and *sbp-1* worms not significantly changed (Fig. 3F), suggesting that the observed PTE effect on reducing fat storage may depend on SBP-1 and SCD. On the other hand, like WT (Fig. 1B), *KQ377* worms that overexpress *sbp-1* had significantly reduced fat content treated by PTE (Fig. 3G), which was consistent with the reduction of SBP-1::GFP expression by PTE (Fig. 3A).

### PTE inhibited food uptake

**Fig 4:**
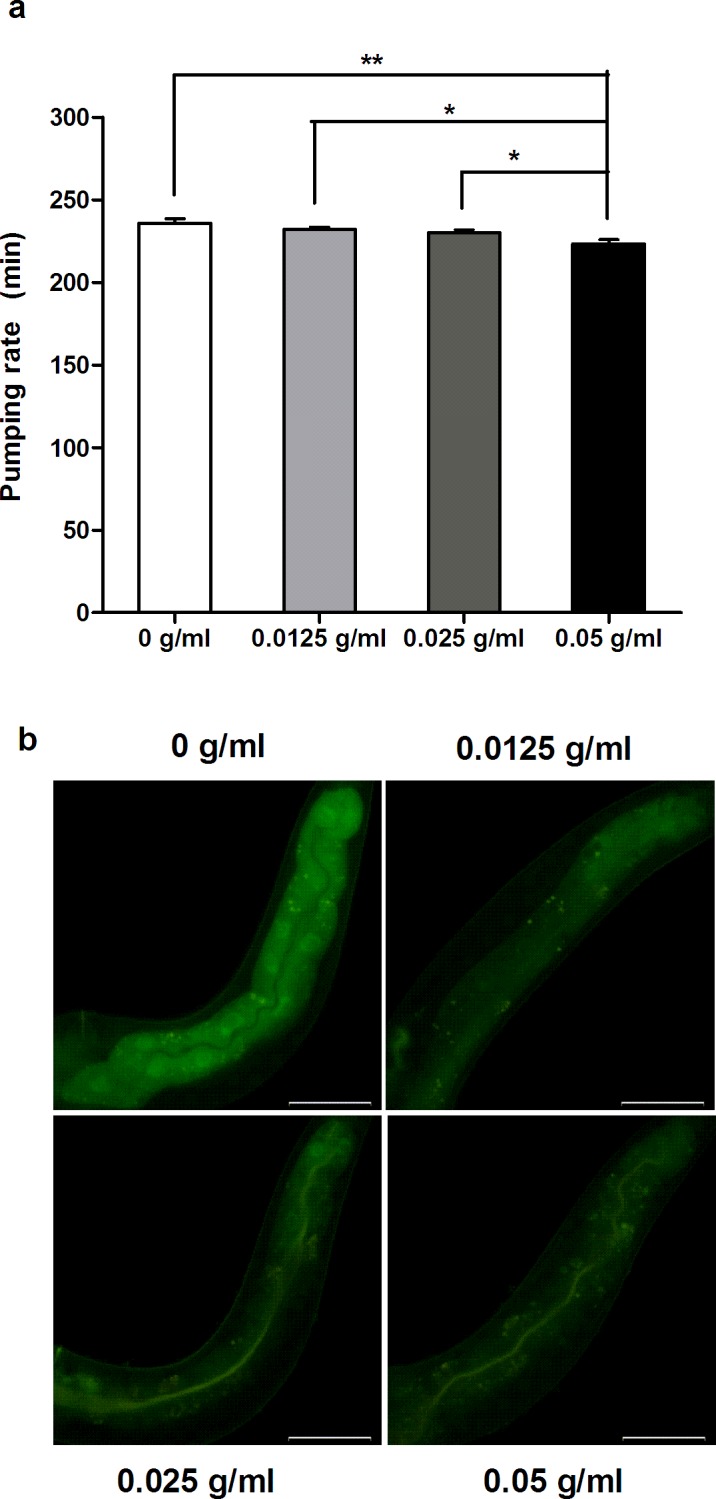
PTE inhibited food uptake. (a) The pumping rate of pharynx was gradually decreased with the increased concentrations of PTE. Data were the mean ± SEM of 12 worms. (*: P<0.05, **: P<0.01). (b) PTE inhibited the uptake of *E*. *coli* food indicated by GFP intensity (∼80% worms showed consistent reduction of expression, around 50 worms totally observed in three biological replicates). Anterior is in left, posterior is in right. Scale bar is 50 um.

One well-known approach to cutting weight and fat storage is to reduce food uptake, but it is not clear if PTE may have an effect on this regard. Though there was no direct indicator this may be the case, we wanted test this possibility. We counted the pumping rate of *C*. *elegans* pharynx treated with different concentrations of PTE, and surprisingly found that the pumping rate indeed decreased with administration of increased PTE concentrations (Fig. 4A). Furthermore, fluorescence testing of the intestine also showed an interesting effect of PTE administration. We fed *E*.*coil* transformed with GFP to several groups of *C*. *elegans*, and among the control group we found that the fluorescence was strongly diffused in whole intestine, but with increasing concentrations of PTE, the fluorescence intensity gradually weakened. Moreover, at high concentration of PTE, fluorescence collected around intestine lumen (Representative samples were shown in Fig. 4B), indicating that Pu-erh tea inhibited food uptake and absorption among *C*. *elegans*.

### PTE affected brood size, growth rate, and lifespan

**Fig 5:**
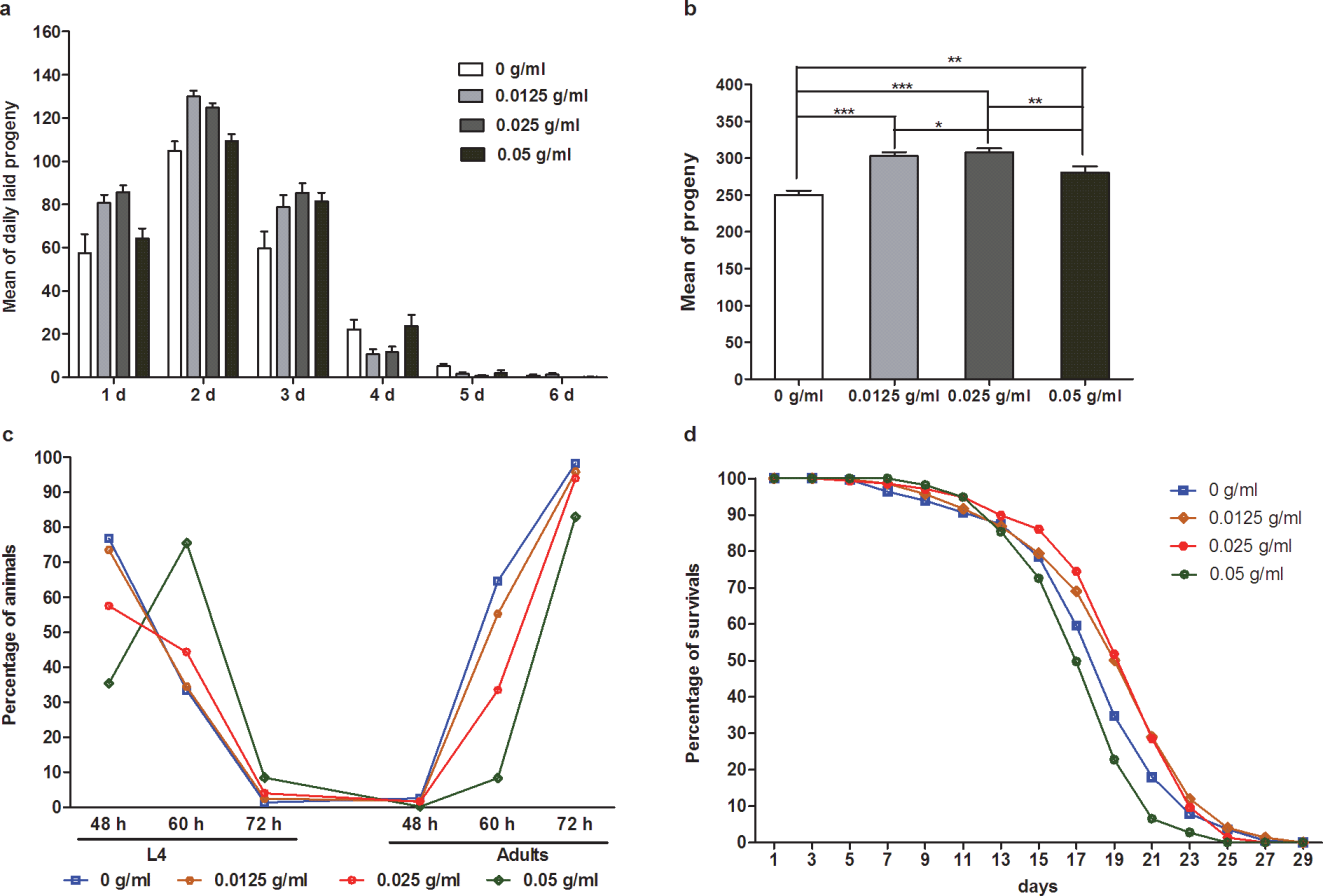
PTE affected growth, brood size, and lifespan. (a) Mean of daily laid progeny (n = 20 worms for each treatment). (b) Mean of progeny (n = 20 worms for each treatment). (c) Growth rate, n = 442, 560, 496, and 620 worms for 0 g/ml, 0.0125 g/ml, 0.025 g/ml,0.05 g/ml, respectively. (d) Lifespan, n = 189, 287, 303, and 266 worms for 0 g/ml, 0.0125 g/ml, 0.025 g/ml,0.05 g/ml, respectively.

PTE contains a large number of bioactive compounds that may have either benefit or adverse effects on *C*. *elegans* in terms of brood size, growth rate and lifespan, but these are almost entirely unknown. Our testing showed that PTE administered at low concentration (0.0125 g/ml) or high concentration (0.05 g/ml) showed no effect on the number of progeny laid daily (Fig. 5B), but both had a slight but significant effect on increased brood size (Fig. 5B). High concentration of PTE (0.05 g/ml) was also found to slow the growth rate of *C*. *elegans* at the early stage (60 h), with the worms successfully reached adulthood after 72 h (Fig. 5C), but no administered concentration seemed to significantly extend or shorten lifespan (Fig. 5D). Taken collectively, these data suggest that administering PTE does not result in any harmful effects for *C*. *elegans*.

## Discussion

PTE was previously reported to inhibit the expression of key genes involved in the biosynthesis of fat. Stearoyl-CoA desaturase (SCD) introduces a double bond between C9 and C10, converting saturated fatty acids (C16:0 and C18:0) to monounsaturated fatty acids (C16:1n-7 and C18:1n-9)[38], which are key substrates for biosynthesis of triglycerides, phospholipids, cholesterol esters, etc. Furthermore, the expression of *scd* genes and the activity of SCD were found to be related to several metabolic diseases[38,42]. PTE was also reported to decrease the expression of SCD-1 mRNA in adipose tissue among rats[44]. However, several factors are known to affect the mRNA expression of *scd* that may or may not eventually reflect the activity of SCD[38,42,45], which is basically measured by the ratio of monounsaturated fatty acids to saturated fatty acids. Our present results showed that the mRNA expression of *fat-5*, *fat-6*, and *fat-7* was reduced by PTE (Fig. 3B), further reducing their translational levels (Fig. 3A). In this study, we noted that introducing PTE slightly but significantly decreased the ratio of oleic acid (C18:1n-9) to stearic acid (C18:0) (Fig. 2B), but only in triglycerides (Fig. 2D). Consistently, the fat content of *fat-6;fat-7* mutants did not decreased by PTE (Fig. 3D, 3E and 3F), further suggesting that PTE reduced fat storage via SCD.

Several recent studies found that PTE down-regulated the expression of transcription factors SREBP-1c[46] and PPARγ[47], which both regulate the expression of *scd-1*. In *C*. *elegans*, *nhr-49* with sequence similarity to PPAR[48], *nhr-80*[32], and *sbp-1* (homolog of SREBP)[20,30,41], regulate the expression of *scd* gene *fat-5*, *fat-6*, and *fat-7*. Mutation of *nhr-49*[48], *nhr-80*[32], or *sbp-1*[30] all resulted in a decreased ratio of oleic acid (C18:1n-9) to stearic acid (C18:0). Here, we found that PTE only inhibited the expression of *sbp-1* (Fig. 3B) and SBP-1::GFP (Fig. 3A), but not *nhr-49* and *nhr-80* (Data not shown). We previously reported that the *sbp-1(ep79)* mutant displayed many phenotypes very like that of *fat-6;fat-7* mutants[30], and in this study we consistently observed that the *sbp-1(ep79)* mutant was also not responsive to PTE induced fat storage reduction (Fig. 3D, 3E and 3F). However, what compound(s) in PTE may directly or indirectly inhibit the expression of *sbp-1* are unknown. Since SBP-1 regulates the transcription of *fat-6* and *fat-7*[20,30,41], inhibition of *sbp-1* by PTE appears to lead to decreased expression of *fat-6* and *fat-7* mRNA (Fig. 3A and 3B), further reducing FAT-7::GFP (Fig. 3A) as well as C18:1n-9/C18:0 (Fig. 2B and 2D).

Acetyl-CoA carboxylase (ACC) and fatty acid synthase (FAS) respectively catalyze the first and second step in *de novo* fatty acid biosynthesis, and *acc* and *fas* are targets of SREBP. Several lines of evidence have demonstrated that PTE down-regulates the mRNA and protein expression of *fas*[15,46,49] but our results showed that the expression of *pod-2* and *fasn-1* encoding fatty acid synthase in *C*. *elegans* was unchanged by PTE (Fig. 3C), which was not surprising since only 7.2% of C16:0 is *de novo* synthesized in *C*. *elegans*[50]. Additionally, consistence with a previous report[46], the expression of *pod-2* was also not changed by PTE (Fig. 3C).

Loss of SCD in either mice[51] or *C*. *elegans*[31] results in the promotion of fat β-oxidation. Since PTE inhibits *fat-5*, *fat-6*, and *fat-7* (Fig. 3B), we used QPCR to investigate the expression of genes predicted to be involved in the mitochondrial, peroxisomal b-oxidation pathways, and lipases. Consistent with the earlier finding by Brock et al.[31], the presence of PTE significantly increased the expression of *acs-2* (S2A Fig.) encoding acyl-CoA synthetase and *ech-1* (S2C Fig.) encoding a trifunctional b-oxidation. Totally, the expression of 13 genes was up-regulated while the expression of 15 was down-regulated by PTE (S2A, S2B, S2C, S2D and S2E Fig.), suggesting that PTE affects the expression of lipolysis genes. Pu-erh tea was also suggested to affect the expression of PI3K/AKt[47,49,52] and AMPK[52,53] signaling pathways that may eventually regulate fat lipogenesis or lipolysis. Curiously though, in our study using *C*. *elegans* as a model, all mutants of *daf-2* encoding insulin/IGF-1 receptor, *age-1* encoding PI3K, *akt-1*, *aa2* encoding AMPK were not resistant to the effects of PTE on fat storage reduction (Data not shown), suggesting that the underlying mechanisms of PTE’s fat storage reduction may not depend on PI3K/AKt and AMPK in *C*. *elegans*.

**Fig 6:**
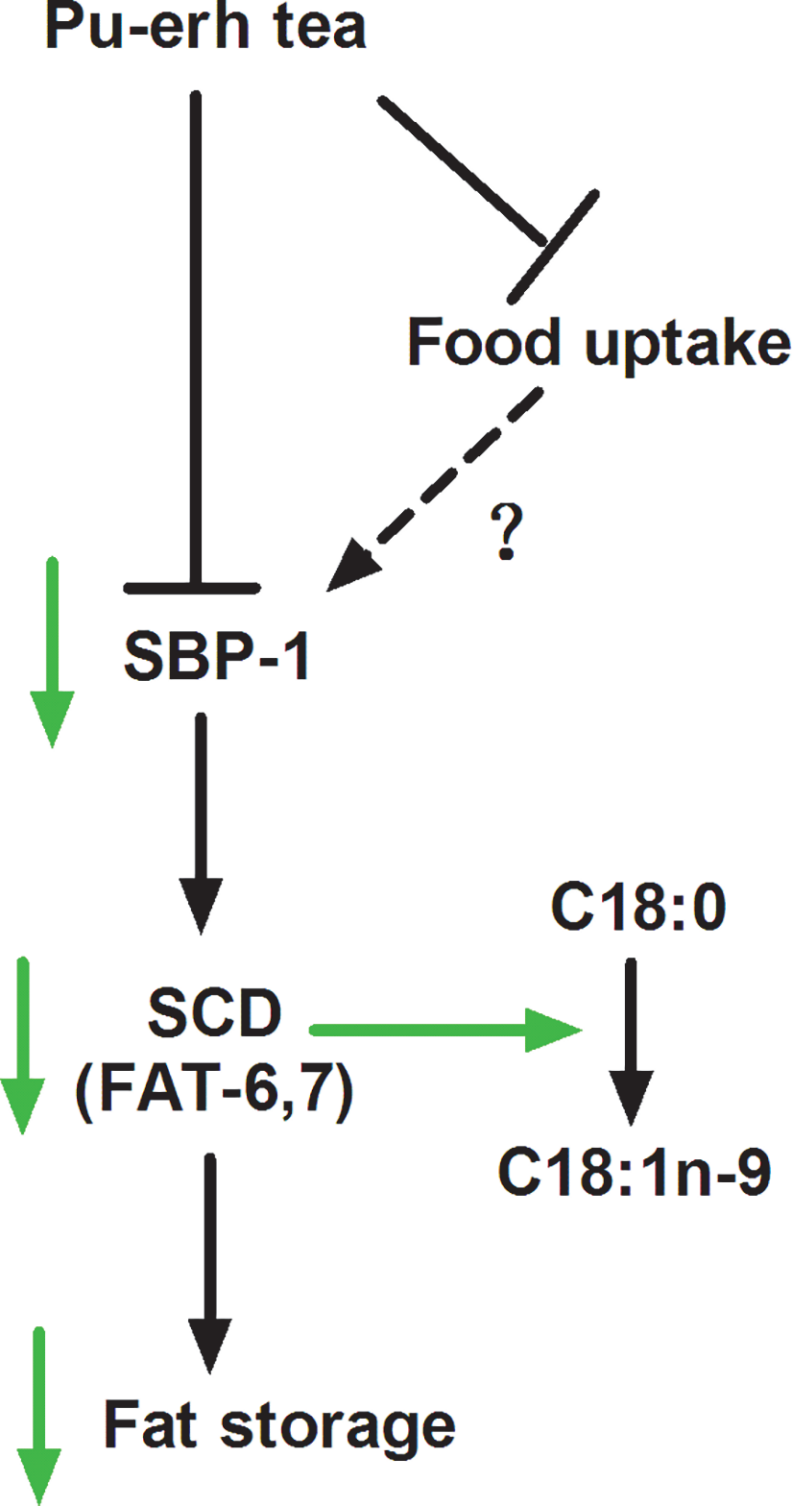
A model of Pu-erh tea reducing fat storage. Pu-erh tea down-regulates the pumping rate, food uptake, as well as the expression of SBP-1 and SCD that reduces the conversion of stearic acid (C18:0) to oleic acid (C18:n-9), eventually reduces the fat accumulation in *C*. *elegans*. Green arrow indicated reduction. Dashed arrow indicated unclear relationship.

Despite variations, anti-obesity compounds or drugs primarily function by inhibiting food uptake, digestion, and the balance of fat synthesis and oxidation. For *C*. *elegans*, feeding depends on the action of the pharynx, a tubular pump responsible for bringing in bacterial nutrients, concentrating them, and grinding them up[54]. The nutrients are further passed through the body, digested, converted, and stored as fat. To date, there has been no clear evidence confirming PTE reduces food uptake either in human and rodent model, though there is some data supporting that PTE may inhibit the activity of pancreatic lipase[46]. In this study, we found that PTE reduced the pumping rate of the pharynx and the digestion of *E*. *coli* transformed with a GFP marker (Fig. 4A and 4B), suggesting that PTE inhibits food uptake in *C*. *elegans*. However, what compound in PTE and signaling pathway responding to PTE inhibition of food uptake was unclear that needed to be further characterized in the future. Most of *C*. *elegans* C18:0 (∼87%) is elongated from the dietary C16:0[50], and is further desaturated to C18:1n-9 by FAT-6 and FAT-7[31,35]. Inhibition of food uptake by PTE may or may not reduce the expression of SBP-1, further down-regulation the expression of SCD to decrease the fat storage in *C*. *elegans* (Fig. 6).

Pu-erh tea is a post-fermentated tea. Several research groups have consistently reported that during fermentation, the levels of catechins epigallocatechin-3-gallate, epigallocatechin, epicatechin-3-gallate, and quinic acid were significantly decreased, but the overall content of gallic acid was elevated[2,55,56]. Gallic acid has been considered one of main active constituents in Pu-erh tea that may explain some of its effect in terms of anti-obesity, hyperglycemia, hyperlipidemia, among others[12,47,57–61]. Here, we found largely consistent results with the earlier reports, providing evidence of how Pu-erh tea can decrease fat storage in *C*. *elegans* (Fig. 1A and 1B). However, we did not find that gallic acid (0.5, 1.0, and 1.5 mg/ml) could be the agent responsible for decreases in fat storage in *C*. *elegans*; quite the contrary, we noted that high concentration of gallic acid (2.0 mg/ml) led to larvae arrest of *C*. *elegans* (Data not shown). This discrepancy may be the difference of animal models, or some unclear factors needed to be further clarified, but the lack of evidence to answer this question only underscores the need to further characterize the various factors and compounds in Pu-erh, especially those responsible for storage and down-regulation of the expression of SCD and SREBP.

## Supporting Information

S1 Fig Quantification of the water extract of Pu-erh tea (PTE). (a) PTE showed a peak at 590 nm using a microplate reader (Biotek, Synergy H1). (b) Diluted water extract of Pu-erh tea (0.05 g/ml, 0.025 g/ml, 0.0125 g/ml, and 0.00625 g/ml) presented a linear relationship at 590 nm wavelength (R2 = 0.9155). (c) Preparation of nematode growth medium (NGM) with different diluted PTE. Data were presented as mean ± SEM of three and four independent experiments.
(DOCX)

S2 Fig Expression of lipid metabolic genes treated by PTE. PTE affected the expression of genes predicated to involve into fatty acid modification/transport, mitochondrial β-oxidation, peroxisomal β-oxidation and lipases. Data were presented as the mean ± SEM of three or four independent biological replicates (*: P<0.05, **: P<0.01, ***: P<0.001).
(DOCX)
