# Supplementary material for: Pu-Erh Tea Down-Regulates Sterol Regulatory Element-Binding Protein and Stearyol-CoA Desaturase to Reduce Fat Storage in Caenorhaditis elegans
Source: PLoS One. 2015 Feb 6;10(2):e0113815. doi: 10.1371/journal.pone.0113815 (PMC4319740; doi:10.1371/journal.pone.0113815)
Supplement: S1 Fig — (a) PTE showed a peak at 590 nm using a microplate reader (Biotek, Synergy H1). (b) Diluted water extract of Pu-erh tea (0.05 g/ml, 0.025 g/ml, 0.0125 g/ml, and 0.00625 g/ml) presented a linear relationship at 590 nm wavelength (R2 = 0.9155). (c) Preparation of nematode growth medium (NGM) with different diluted PTE. Data were presented as mean ± SEM of three and four independent experiments. (DOCX) [file pone.0113815.s001.docx]

**Figure S1.** **Quantification of the water extract of Pu-erh tea (PTE).** (a) PTE showed a peak at 590 nm using a microplate reader (Biotek, Synergy H1). (b) Diluted water extract of Pu-erh tea (0.05 g/ml, 0.025 g/ml, 0.0125 g/ml, and 0.00625 g/ml) presented a linear relationship at 590 nm wavelength (R^2^=0.9155). (c) Preparation of nematode growth medium (NGM) with different diluted PTE. Data were presented as mean ± SEM of three and four independent experiments.
