# Supplementary material for: Pu-Erh Tea Down-Regulates Sterol Regulatory Element-Binding Protein and Stearyol-CoA Desaturase to Reduce Fat Storage in Caenorhaditis elegans
Source: PLoS One. 2015 Feb 6;10(2):e0113815. doi: 10.1371/journal.pone.0113815 (PMC4319740; doi:10.1371/journal.pone.0113815)

**Figure S2. Expression of lipid metabolic genes treated by PTE.** PTE affected the expression of genes predicated to involve into fatty acid modification/transport, mitochondrial β-oxidation, peroxisomal β-oxidation and lipases. Data were presented as the mean ± SEM of three or four independent biological replicates (*: P<0.05, **: P<0.01, ***: P<0.001)


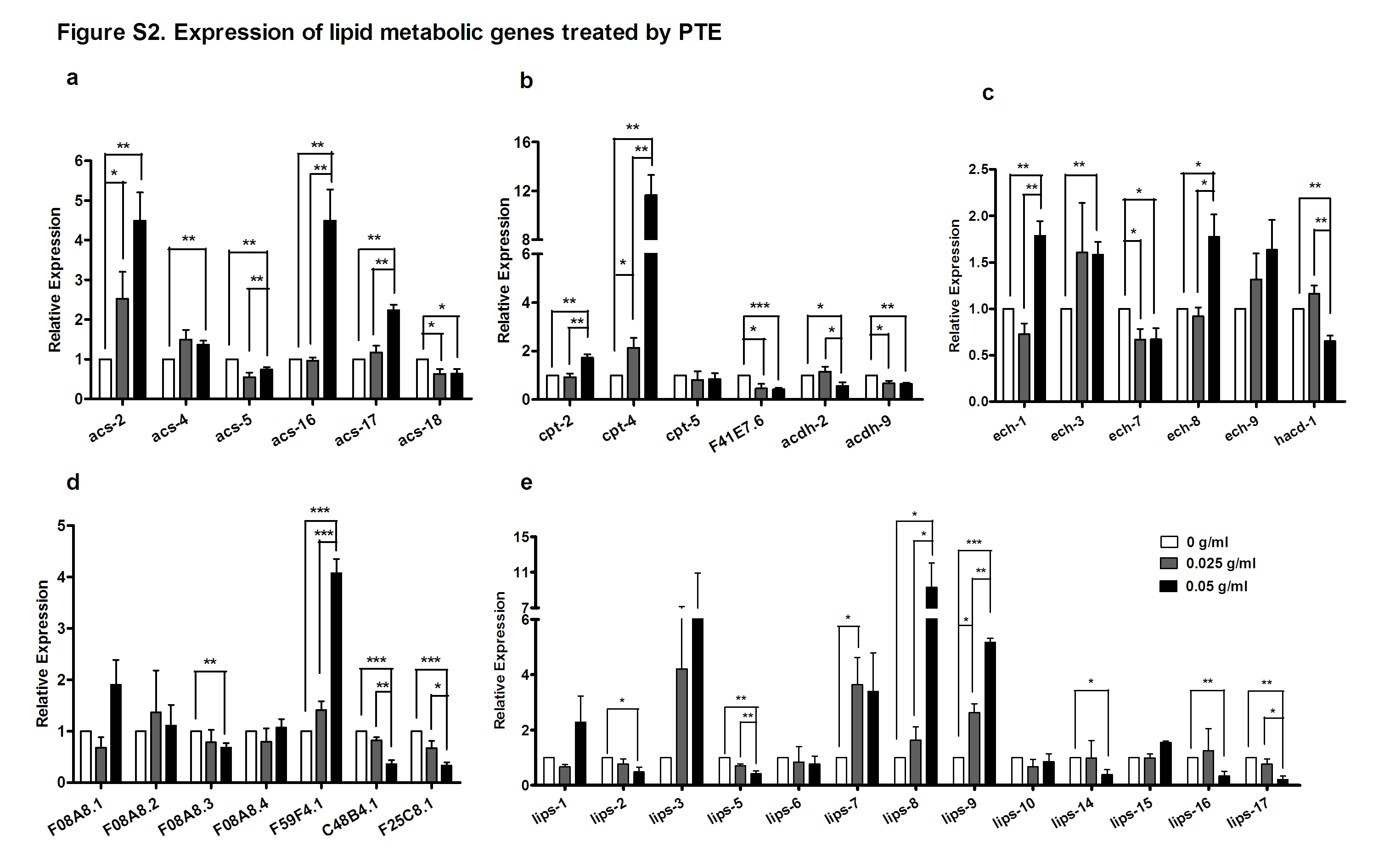

Supplement: S2 Fig — PTE affected the expression of genes predicated to involve into fatty acid modification/transport, mitochondrial β-oxidation, peroxisomal β-oxidation and lipases. Data were presented as the mean ± SEM of three or four independent biological replicates (*: P<0.05, **: P<0.01, ***: P<0.001). (DOCX) [file pone.0113815.s002.docx]
